# A Huge Lymphangioma Mimicking Pleural Effusion with Extension to Both Chest Cavities: A Case Report and Review of Literature

**Published:** 2015-03

**Authors:** Mohsen Sokouti, Nassir Rostambeigi, Monireh Halimi, Seyed Ziaeddin Rasihashemi

**Affiliations:** 1 Department of Cardiothoracic Surgery, Tabriz University of Medical Sciences, Tabriz, Iran;; 2 Department of Radiology, University of Minnesota, USA;; 3 Department of Pathology, Tabriz University of Medical Sciences, Tabriz, Iran

**Keywords:** Lymphangioma, Mediastinum, Surgery, Pleural effusion

## Abstract

Mediastinal lymphangioma is primarily a benign lesion and the majority of the cases are found incidentally. These lesions account for approximately 1% of all mediastinal tumors. Here we present a giant mediastinal cystic mass in a 35-year-old female who was presented with severe respiratory distress. On the plain chest radiography and CT scan, a massive left pleural effusion with large parasternal and mediastinal lymphadenopathy was seen. Thoracentesis was performed and 400 cm³ of clear fluid was drained from the left hemithorax. However, a subsequent CT scan with contrast and the same technique 40 days later showed a large cystic mass in the mediastinum protruding to the right and left hemi thoraces. The giant cystic mass was resected via right and left anterior thoracotomies. Histopathological examination revealed a diagnosis of lymphangioma. The patient has been alive and without tumor recurrence and has been followed for 2 years.

## Introduction

Mediastinal lymphangioma is a slow growing mass with benign features, and account for 1% of all mediastinal tumors.^1^ They are usually located in the neck or axillary regions, but mediastinal location is seen in about one percent of the cases.^2^ Lymphangiomas are rare congenital malformations consisting of focal proliferations of well-differentiated lymphatic tissue in multi cystic or sponge like structures.^2^ Lymphangioma is usually asymptomatic due to its soft consistency but compression of adjacent structures can be seen due to the mass effect of a large tumor. The mean age group at diagnosis is reported to be around 36 years, with a male to female ratio of 1 to 3.^3^ Preoperative diagnosis is usually difficult due to non-specific appearance on imaging studies and most of the cases are diagnosed during or after the operation. It is however important to suspect this condition as these lesions appear to have an excellent prognosis after complete resection of the tumor.^3^^-^^5^ Here we present a giant mediastinal lymphangioma with extension into both sides of hemithorax which has not been previously reported in the literature.

## Case Presentation

**Figure 1:**
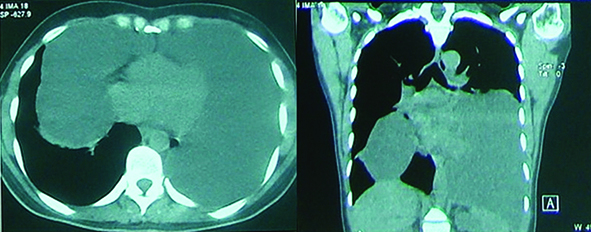
Sagital and coronal sections of chest CT scan showing huge mediastinal lymphangioma protruding into both chest cavities.

A 35-year-old woman who presented with severe dyspnea and sweating was admitted to our tertiary referral hospital in March 2009. Her chief complaint was severe worsening dyspnea. Plain chest radiography and CT scan demonstrated huge parasternal lymphadenopathy and a massive left pleural effusion (figure 1). She underwent left thoracentesis in another facility and 400 cm³ of clear fluid was aspirated and sent for biochemistry and cytologic examinations.

She was referred to our facility for further work-up. An informed consent was obtained from the patient in order to use the clinical data and imaging studies for research purposes. In our hospital a second contrast-enhanced chest CT scan and the same techniques was performed 40 days after the initial CT scan. A giant cystic mass was found in the mediastinum extending to both hemithoracis measuring (35×25×9 cm). She was subsequently prepared for urgent surgical intervention.

**Figure 2:**
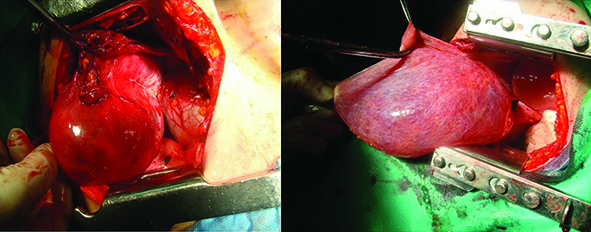
Right and left thoracotomy showing views of huge cystic mediastinal lymphangioma in the anterior mediastinum protruding into both hemithoracis.

In the right anterior thoracotomy, a right cystic mass (10×9×8 cm) was identified protruding to the anterior mediastinum and left hemithorax. Left anterior thoracotomy was also performed for resection of the left sided component of this large cystic mass (20×15×10 cm) extending from the anterior mediastinum to the left hemithorax (figure 2). There were no pleural effusions in both exploratory thoracotomies. Cystic masses adhering to the left and right lower horns of thymus were resected without any damage to phrenic nerves. Chest tubes were removed on postoperative day 4 and the patient was discharged on postoperative day 6 with no postoperative complication.

**Figure 3:**
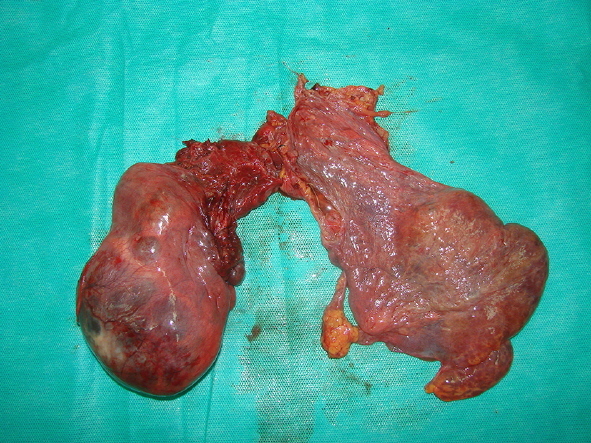
Macroscopic features of the huge cystic mediastinal lymphangioma of anterior mediastinum.

**Figure 4:**
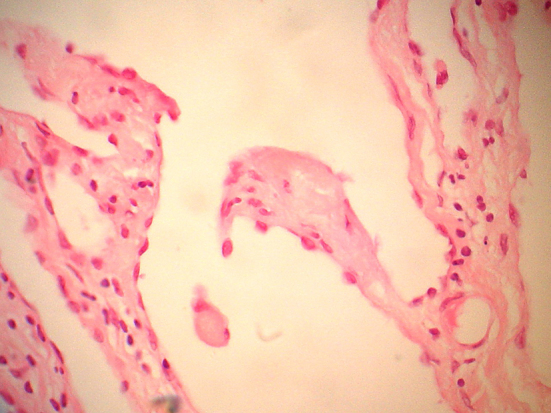
The mediastinal lymphangioma is made of up of massively dilated cystic lymphatic spaces lined by endothelial cells and separated by intervening connective tissue stroma.

On gross pathology, the mass was demonstrated as a brown-gray colored lesion measuring (35×25×9 cm). Gross pathologic examination of specimens showed a membranous appearance with cystic areas filled with clear fluid (figure 3). The microscopic histopathology revealed variable size cystic lesions lined by flat endothelium and fragments of thymus tissue. There was no evidence of capsular invasion or malignant features such as atypical nuclei or mitotic figures (figure 4). The operation was uneventful. The patient has been followed in our thoracic surgery clinic and no recurrence has been observed in two years later after surgery.

## Discussion

Cystic lesions of the mediastinum are generally classified as a congenital developmental malformation with involvement of the lymphatic tissues.^4^ Benign cystic lymphangioma is usually localized in the neck (75%), axila (20%) or rarely in the mediastinum (1%).^6^ Mediastinal lymphangioma is clinically asymptomatic in the majority of cases and it is mostly an incidental finding. Giant mediastinal cysts however may produce respiratory symptoms as was present in our patient. Lymphangiomatic lesions are classified into three types: cystic (macro cyst type, hygroma), simple (super micro cystic type, capillary), and cavernous (micro cystic type).^4^

On chest radiographs, lymphangiomas appear as well-defined, round, lobular masses.^2^^,^^4^ CT scan provides helpful information in regard to size, density, and site of the cysts.^7^ The most common finding on CT scan is usually a homogeneous non-enhancing thin walled unilocular mass. They usually appear as low attenuating masses similar to water, although they can have higher attenuation if there is a combination of fluid, solid tissues, or fat. A multi-septal, loculated mass may also be seen sometimes on CT scan or MRI, which can make the diagnosis of lymphangioma particularly challenging.^5^ MRI is a useful diagnostic tool in preoperative diagnosis and can differentiate different types of lymphangioma.^6^ At MRI, the lesions present as heterogeneous signal intensities on T1-weighted images and high signal intensity on T2-weighted images.^2^^,^^8^ Siegle and Pilla indicated that at MRI, lymphangioma can have different signal intensities which may make the diagnosis sometimes difficult. Differential diagnosis of mediastinal lymphangioma includes cystic teratoma and cystic thymoma.^4^

Complications of untreated lymphangioma include infection, chylothorax and chylopericardium. In addition, unilateral or bilateral pleural effusions, which are often chylous, may be present.^2^ Although no explanation has been given for the mechanism by which mediastinal lymphangioma can protrude into hemithoraces, it is probably similar to the way giant thymolipomas are hanging from the anterior mediastinum and eventually protrude to hemithoraces.^9^

Bossert et al. briefly reported a large mediastinal lymphangioma with similar clinical presentation and with protrusion into left mediastinum.^6^ They indicated that, but not similar to the size of the giant mediastinal lymphangioma in this patient. The report of lymphangioma of this size has not been previously reported in the literature**. **This case shows the importance of timely diagnosis and the imaging findings that can be misleading in the initial encounters, but adequate follow up can reveal the accurate diagnosis. A high index of clinical suspicion may be generated by relevant imaging findings and rule out of other etiologies.

Surgical management is the treatment of choice, although surgery can become at times challenging due to the mass being adherent to large vessels of mediastinum.^7^^,^^10^ After complete resection, the prognosis of this disease appears to be good.^10^ Nevertheless, complete surgical resection of lymphangiomas needs to be followed up by imaging to detect any recurrence.^2^ In conclusion, mediastinal lymphangioma may present as a huge cystic lesion with mass effect but proper diagnosis and surgical management is often followed by favorable prognosis.
